# *L,L*-Diaminopimelate Aminotransferase from Chlamydomonas reinhardtii: A Target for Algaecide Development

**DOI:** 10.1371/journal.pone.0020439

**Published:** 2011-05-25

**Authors:** Renwick C. J. Dobson, Irma Girón, André O. Hudson

**Affiliations:** 1 Department of Biochemistry and Molecular Biology, Bio21 Molecular Science and Biotechnology Institute, The University of Melbourne, Parkville, Victoria, Australia; 2 Biomolecular Interaction Centre, School of Biological Sciences, University of Canterbury, Christchurch, New Zealand; 3 School of Biological Sciences, Rochester Institute of Technology, Rochester, New York, United States of America; University Paris Diderot-Paris 7, France

## Abstract

In some bacterial species and photosynthetic cohorts, including algae, the enzyme *l,l*-diaminopimelate aminotransferase (DapL) (E.C. 2.6.1.83) is involved in the anabolism of the essential amino acid *L*-lysine. DapL catalyzes the conversion of tetrahydrodipicolinate (THDPA) to *l,l*-diaminopimelate (*l,l*-DAP), in one step bypassing the DapD, DapC and DapE enzymatic reactions present in the acyl DAP pathways. Here we present an *in vivo* and *in vitro* characterization of the DapL ortholog from the alga *Chlamydomonas reinhardtii* (*Cr-*DapL). The *in vivo* analysis illustrated that the enzyme is able to functionally complement the *E. coli dap* auxotrophs and was essential for plant development in Arabidopsis. *In vitro*, the enzyme was able to inter-convert THDPA and *l,l*-DAP, showing strong substrate specificity. *Cr*-DapL was dimeric in both solution and when crystallized. The structure of *Cr*-DapL was solved in its *apo* form, showing an overall architecture of a α/β protein with each monomer in the dimer adopting a pyridoxal phosphate-dependent transferase-like fold in a V-shaped conformation. The active site comprises residues from both monomers in the dimer and shows some rearrangement when compared to the *apo*-DapL structure from Arabidopsis. Since animals do not possess the enzymatic machinery necessary for the *de novo* synthesis of the amino acid *l*-lysine, enzymes involved in this pathway are attractive targets for the development of antibiotics, herbicides and algaecides.

## Introduction

The essential amino acid *l-*lysine (lys) is anabolized *via* two evolutionary lineages that are divergent in nature. One pathway uses the intermediate α-aminoadipic acid (AAA), which occurs in yeast, fungi and in some species belonging to the kingdom archaea [1], [2]. The alternative pathway utilizes the intermediate diaminopimelic acid (DAP) and is present in most bacterial species and photosynthetic cohorts.

To date, four variants of the DAP/lys pathway have been identified: the two acyl pathways, which utilize succinylated or acetylated intermediates; the *meso*-diaminopimelate (*meso-*DAP) dehydrogenase (Ddh) pathway, which has been identified in only a few species thus far; and the recently discovered *l,l-*diaminopimelate (*L,L*-DAP) aminotransferasae (DapL) pathway [3], [4], [5], [6].

**Figure 1:**
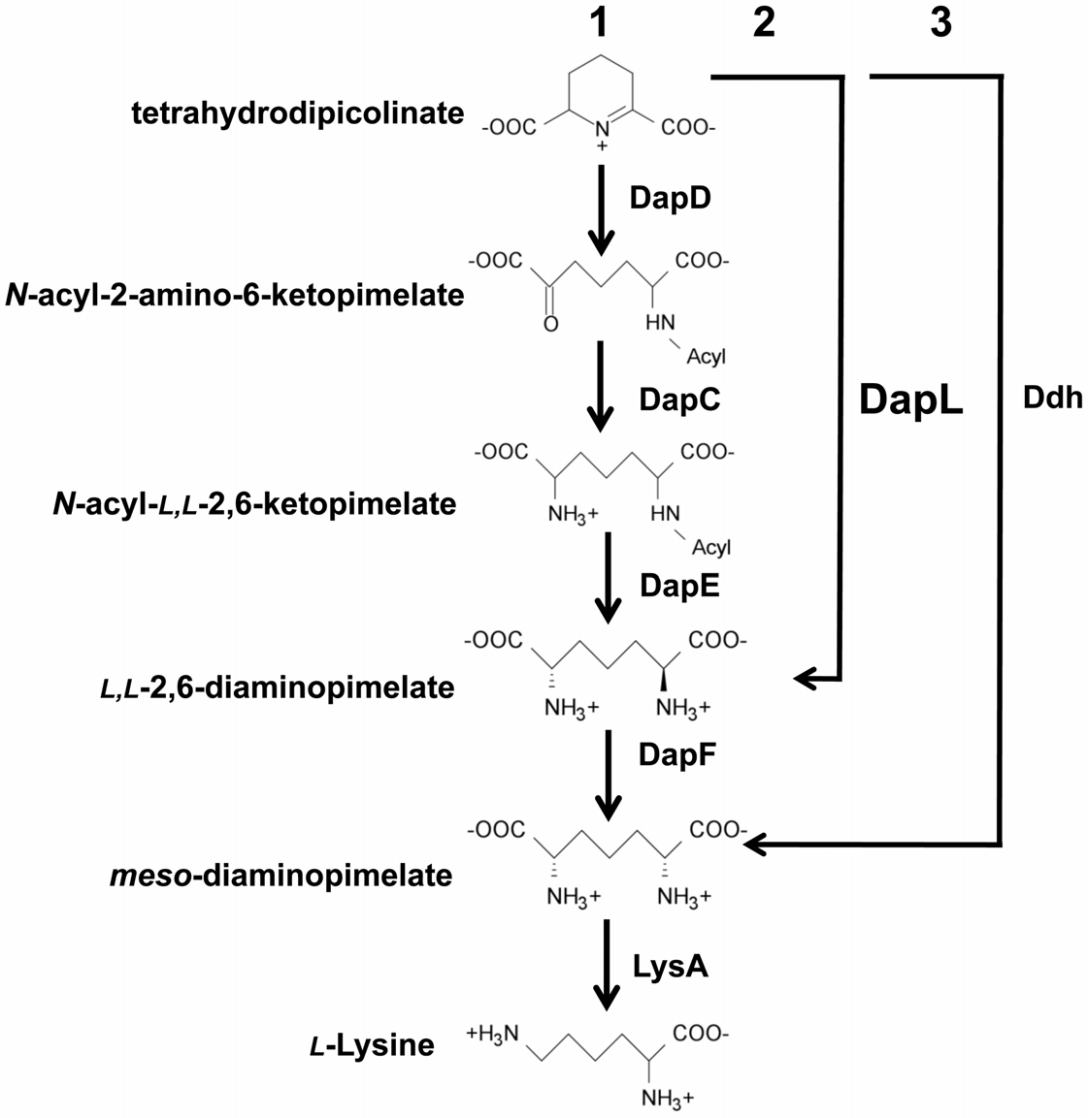
DAP/*L*-lysine anabolic pathways. The pathways are denoted by the acyl pathways (1), *l,l*-diaminopimelate aminotransferase pathway (2) and the *meso-*DAP dehydrogenase pathway (3). The abbreviations of the enzymes are as follows: tetrahydrodipicolinate acylase (DapD), acyl-amino-ketopimelate aminotransferase (DapC), acyl-ketopimelate deacylase (DapE), diaminopimelate epimerase (DapF), diaminopimelate decarboxylase (LysA), *m*-diaminopimelate dehydrogenase (Ddh) and *l,l*-diaminopimelate aminotransferase (DapL).

The DAP/lys pathway can be divided into three parts. The first part of the pathway is the synthesis of tetrahydrodipicolinate (THDPA) from the amino acid *l-*aspartate (Figure 1). This feature is common to all four variants. The conversion from *l*-aspartate to THDPA is facilitated by a series of reactions carried out by the enzymes LysC, AsD, DapA, and DapB respectively [4]. The second and central portion of the pathway, comprising the conversion of THDPA to the penultimate intermediate *meso-*DAP, distinguishes the four variants. In the acyl pathways, four enzymes are needed for this conversion to occur. These reactions are carried out by the enzymes: DapD, DapC, DapE and DapF, respectively (Figure 1). In the Ddh pathway, THDPA is converted to *meso-*DAP by the enzyme *meso*-diaminopimelate dehydrogenase (Ddh), in one step bypassing the DapD, DapC, DapE and DapF reactions [7].

Hudson *et al.* recently discovered the *l,l*-diaminopimelate (*l,l*-DAP) aminotransferase pathway[4]. In this pathway, *l,l*-DAP is synthesized from THDPA in one step, using the amino acid *l*-glutamate as an amino donor and THDPA as the amino acceptor, bypassing the DapD, DapC and DapE enzymatic steps that are present in the acyl pathways (Figure 1).

The third and final step in the DAP/lys pathways is the conversion of *meso-*DAP to *l*-lysine and is catalyzed by the enzyme *meso*-diaminopimelate decarboxylase (LysA). This enzymatic reaction is also common to all four DAP/lys anabolic variants (Figure 1).

The various DAP/lys biosynthetic pathways are presumed validated targets for the design of antibiotics and herbicides [8], [9]. *meso-*DAP is one of the cross-linking amino acids in the cell wall of Gram-positive bacteria and *l*-lysine plays the same role in Gram-negative bacteria [10]. Compounds that inhibit enzymes involved in the DAP/lys pathways are of interest since animals are unable to carry out the synthesis of DAP/lys *de novo*. From a bacterial point of view, inhibiting the pathway would eventually lead to peptidoglycan lysis due to osmotic pressure followed by cell death [8], [11]. From a plant/photosynthetic cohort point of view, the inhibition of the DAP/lys pathway would be detrimental to the organism, since it would be unable to synthesize *l*-lysine necessary for protein synthesis. Therefore, enzymes affiliated with this pathway are very attractive targets for antibacterial, herbicide and algaecide development.

The structure of DapL from *Arabidopsis thaliana* was recently reported [12], [13]. DapL enzymes can be classified into two groups based on sequence similarity: Type I enzymes originate from plants and Chlamydia, while Type II enzymes, which share about 30% identity, are primarily found in some bacteria [3]. Based on ligand bound structures, the binding modes for the substrates have been detailed and such structural detail will be useful for inhibitor design [13]. Indeed, inhibitors for the *A. thaliana* enzyme have already been reported [14].

We are also interested in designing inhibitors of enzymes in the *l*-lysine biosynthetic pathway [15], [16], [17], [18] based primarily on our knowledge of enzyme function and structure [19], [20], [21], [22], [23], [24]. Here we identify and characterize the first Type I *l,l*-DAP aminotransferase ortholog from an algae, *Chalmydomonas reinhardtii*, annotated by the locus tag CHLREDRAFT_129557. We present the crystal structure of the enzyme and show, for the first time, that it is dimeric in solution using analytical ultracentrifugation. In addition, we verify that DapL is essential in the photosynthetic cohort Arabidopsis. The structural and kinetic properties of the algal enzyme will be valuable information for the identification of natural inhibitors or the design of *pseudo*-substrate(s) to facilitate algaecide development.

## Results and Discussion

### Identification of the DapL orthologous gene from *C. reinhardtii*

In order to identify the DapL ortholog from *C. reinhardtii*, the genome of the alga was searched using the Arabidopsis protein annotated by the locus At4g33680 as the query using the BLASTP algorithm (http://www.chalmy.org/cgi-bin/webblast.pl). The search resulted in the identification of the an enzyme annotated as an *L,L*-diaminopimelate aminotransferase by the locus tag CHLREDRAFT_129557 (*Cr*-DapL) that was 65% identical to the Arabidopsis enzyme.

**The gene annotated by the locus tag CHLREDRAFT_129557 encodes** ***L,L*** **-diaminopimelate aminotransferase**

**Figure 2:**
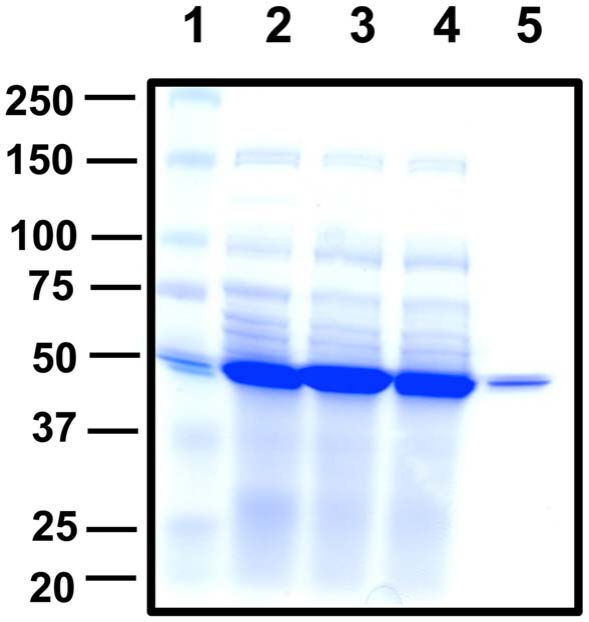
Recombinant expression and purification of *Cr*-DapL from *E. coli.* Lane (1)–Protein marker (kDa), Lane (2)–10 µg uninduced soluble protein extract, Lane (3)–10 µg induced soluble extract, Lane (4)–10 µg of post binding protein, Lane (5)–1.0 µg pure recombinant *Cr*-DapL protein. The proteins were resolved on a SDS-PAGE gel containing 10% (^w^/_v_) acrylamide and the gel was stained using Coomassie Blue.

To assess the function of CHLREDRAFT_129557, the full-length cDNA was cloned and the enzyme was purified to homogeneity using affinity chromatography (Figure 2). The *o*-aminobenzaldehyde (OAB) assay was used to test whether *Cr*-DapL had *L,L*-diaminopimelate aminotransferase activity and to determine the substrate specificity of the enzyme. The results from this analysis illustrate that, like the Arabidopsis enzyme, the algal enzyme is specific for *L,L*-DAP. No enzymatic activity was observed when various other amino donors that are structurally similar to *L,L*-DAP, including the racemic isomer *meso-*DAP (Table 1), were assayed. The same was true for the amino acceptor. Using the same assay, *Cr*-DapL activity was only present when 2-ketoglutarate was used as the amino acceptor. No activity was observed when various 2-oxoacids were used in combination with *L,L*-DAP (Table 1).

**Table 1:** Substrate specificity of *Cr*-DapL using four amino donors.

Amino Donor	Relative Activity (%)	Amino Acceptor	Relative Activity (%)
*l,l*-DAP	100	2-Ketoglutarate	100
*meso-*DAP	0	Pyruvate	0
*l*-Lysine	0	Prephenate	0
*l*-Ornithine	0	Oxaloacetate	0
		Oxovelarate	0

Relative substrate specificity of *Cr*-DapL using various amino donors. The assay measured the production of dihyrdoquinazolium using the OAB assay at 400 nm using 0.5 mM amino donor and 2 mM 2-oxoglutarate. Relative substrate specificity of *Cr*-DapL using various amino acceptors. The assay measured the production of dihyrdoquinazolium using the OAB assay at 400 nm using 2 mM of each acceptor and 0.5 mM of *l,l*-DAP.

### Kinetic properties of *Cr*-DapL

The pure recombinant enzyme was used to perform enzyme assays to assess the kinetic properties using forward and reverse quantitative assays. In the reverse assay, *L,L*-DAP serves as the amino acceptor and 2-ketoglutarate serve as the amino acceptor. In the anabolic direction of *l-*lysine synthesis, glutamate serves as the amino donor and THDPA serves as the amino acceptor. Using these assays, the kinetic properties of the enzyme were tested at varying concentrations of one substrate and at saturation levels of other substrates (Supplementary Figure S1). The reciprocal plots were linear and were consistent with Michaelis-Menten kinetics. The *V*_max_ for the forward and reverse directions were calculated along with the apparent *K*_M_ for the various substrates. The enzyme has a maximum velocity of approximately 11.6 µmol min^−1^ mg^−1^ in the reverse direction and 0.68 µmol min^−1^ mg^−1^ in the forward direction (Table 2). The apparent *K*_M_ for the four substrates were 0.3 mM for *L,L*-DAP, 2.2 mM for 2-ketoglutarate, 0.10 mM for THDPA and 0.9 mM for glutamate.. The kinetic properties of *Cr*-DapL are comparable to the Arabidopsis ortholog that was previously characterized (Table 3).

**Table 2:** Kinetic properties of *Cr*-DapL.

	Assay	*V*_max_	*k*_cat_	Substrate	*K*_M_
		(µmoles min^−1^ mg^−1^)	(s^−1^)		(mM)
*Cr*-DapL	Reverse	11.6±3.2	19.0	*l,l*-DAP	0.3±0.02
				2-ketoglutarate	2.2±0.7
	Forward	0.68±0.2	1.1	THDPA	0.10±0.01
				*l*-glutamate	0.9±0.4
*Ar*-DapL	Reverse	22.3±0.3	17.6	*l,l-*DAP	0.07±0.02
				2-ketoglutarate	8.7±0.3
	Forward	0.38±0.01	0.3	THDPA	0.38±0.04
				*l*-glutamate	1.9±0.4

The quantitative assays used to determine the kinetic parameters for *Cr*-DapL are described in the methods. The *Ar*-DapL kinetic parameters are listed as reported by Hudson, *et al*., 2006.

**Table 3:** Hydrodynamic properties of *Cr-*DapL.

Model	Mass (kDa)	*s*_20,w_ A	*f/fo* B	r.m.s.d.	Runs test-Z score
*c(s)*-distribution	-	5.41	1.51	0.005	3.1
*c(M)*-distribution	100.2^C^	-	1.48	0.005	3.0
Discrete species	99±3^D^	-	-	0.005	5.8

^A^Standardized sedimentation co-efficient taken from the ordinate maximum of the *c(s)* distribution.

^B^Frictional ratio calculated assuming a prolate ellipsoid shape and also assuming a single species [38].

^C^Mass taken from the ordinate maximum of the *c(M)* distribution.

^D^Error reflects the 68.3% confidence interval as implemented in SEDFIT [26], [27].

### *Cr*-DapL is able to functionally complement the *E. coli dapD/dapE* (AOH1) mutant

**Figure 3:**
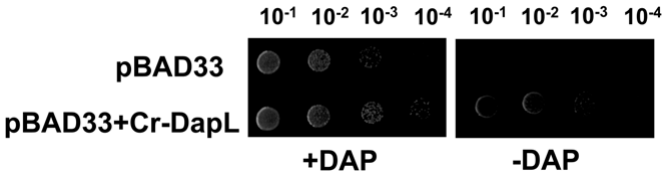
Functional complementation of the *E. coli dapD/E* mutant. Functional complementation was tested using the *E. coli dapD/E* double mutant (AOH1). The plasmids pBAD33 and pBAD33+*Cr*-DapL were selected on LB agar medium supplemented with 50 µg mL^−1^ DAP and 34 µg mL^−1^ chloramphenicol. The bacteria were grown to an OD of 0.5 at 600 nm, the strain were serially diluted to 10^−1^, 10^−2^, 10^−3^ and 10^−4^ using 0.85% (^w^/_v_). The strain harboring the pBAD33 and pBAD33+*Cr*-DapL was replica-plated onto LB medium plus 0.2% (^w^/_v_) arabinose with or without 50 µg mL^−1^ DAP. The cultures were grown at 30 °C for 24 hours.

The *E. coli* mutant AOH1 is suitable for a functional complementation assay because it harbors loss-of-function mutations in *dapD* and *dapE* genes. For this strain, the cells lyse because of osmotic stress, due to the lack of *meso*-DAP as a cross linking amino acid in the cell wall. Thus, the strain is deemed auxotrophic for DAP. The AOH1 strain was transformed with either an empty plasmid or a plasmid expressing *Cr*-DapL. While the mutant is able to grow only on media supplemented with DAP, only the mutant strain expressing the algal enzyme is able to grow on DAP-free media (Figure 3). The results from this assay indicate that the enzyme is able convert THDPA to *L,L*-DAP directly bypassing the DapD, DapC and DapE enzymatic reactions present in the *E. coli* pathway (Figure 1).

### Structure of *Cr*-DapL

To determine the structural properties of the enzyme we employed circular dichroism (CD) spectroscopy to gauge the secondary structure, analytical ultracentrifugation to establish the oligomeric state, and X-ray crystallography to define the macromolecular structure of the enzyme.

**Figure 4:**
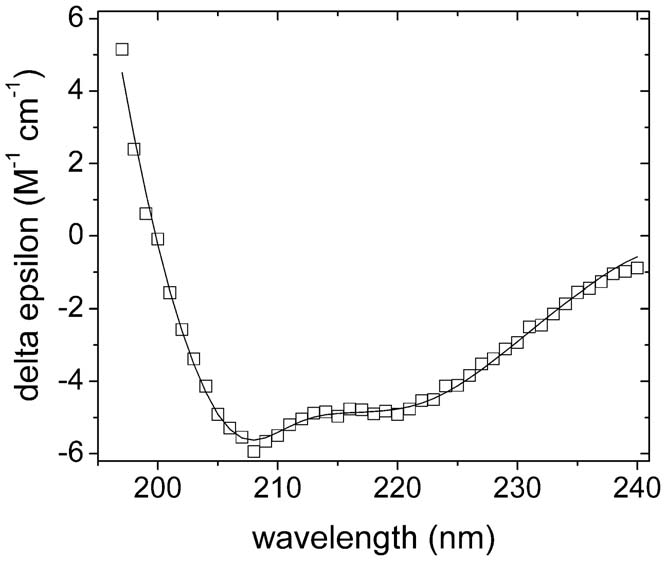
CD analysis of *Cr*-DapL. The wavelength scans were performed between 240 and 195 nm. The scan was performed at a *Cr*-DapL concentration of 1 µM. The final spectrum (□) is the average result from three scans taken at 20°C. The CONTIN algorithm from the CDpro software package produced the best fit (solid line) against the SP43 protein database [25] with an r.m.s.d. = 0.18 M^−1^ cm^−1^. The fit predicts ∼50% α-helix content, ∼15% β-sheet, ∼15% turn, and ∼20% unordered.

CD analysis of *Cr*-DapL resulted in spectra (Figure 4, open symbols) that displayed double minima at approximately 208 nm and 222 nm, suggesting that the enzyme was folded. In order to predict the secondary structure proportions, three algorithms were used from the CDpro software package, CDSSTR, CONTIN and SELCON3, against relevant protein databases. The best fit for the *Cr*-DapL protein (Figure 4, solid line) resulted from using the CONTIN algorithm against the SP43 database [25], which predicted *Cr*-DapL to have predominantly α-helical secondary structure (∼50%), in combination with significant proportions of β-strand (∼15%), unordered structure (∼20%), and turn (∼15%), under the buffer conditions used in this experiment (r.m.s.d. = 0.18 M^−1^ cm^−1^).

To characterize the quaternary structure of *Cr*-DapL in solution, sedimentation velocity studies were employed in the analytical ultracentrifuge at a protein concentration of 9.2 µM. The data acquired for *Cr*-DapL were fitted to a continuous size-distribution model (Table 4) [26], [27]. This yielded a modal sedimentation coefficient (s*_20,w_*) of 5.41 S (r.m.s.d. = 0.005 and Runs test-Z score = 3.1) (data not shown).

**Table 4:** Refinement statistics for the crystal structure of *Cr-*DapL.

Space group	*P*2_1_2_1_2_1_
Unit cell parameters(Å)	*a* = 58.9, *b* = 91.8, *c* = 162.8
(°)	α = β = γ = 90
Refinement resolution (outer shell)	50.0–1.55 (1.59–1.55)
*R*_free_ † ‡ (outer shell)	17.1 (20.0)
*R*_work_ †(outer shell)	12.5 (14.2)
Unique reflections	122,383
Non-H atoms	
Protein	6295
Ligands	47
Solvent (H_2_O)	888
Solvent content (%)	51
Mean isotropic *B* (protein)(Å^2^)	19.0
Side chain	22.2
Main chain	15.3
Mean isotropic *B* (solvent)(Å^2^)	34.9
Mean isotropic *B* (ligands)(Å^2^)	23.9
Residues in Ramachandran plot	
Most favored regions (%)	98.8
Additionally allowed regions (%)	1.1
Disallowed regions (%)	0.1
R.m.s.d. values from ideal geometry	
Bond lengths (Å)	0.026
Bond angles (deg)	1.98
Dihedrals (deg)	6.2

† *R* = ∑||*F*_obs_ *|*-|*F*_cal_||/∑*|F*_obs_ *|*, where *F*_obs_ and *F*_cal_ are the observed and calculated structure-factor amplitudes, respectively.

‡ *R*_free_ was calculated with 2.1% of the diffraction data and was selected randomly and omitted from the refinement.

**Figure 5:**
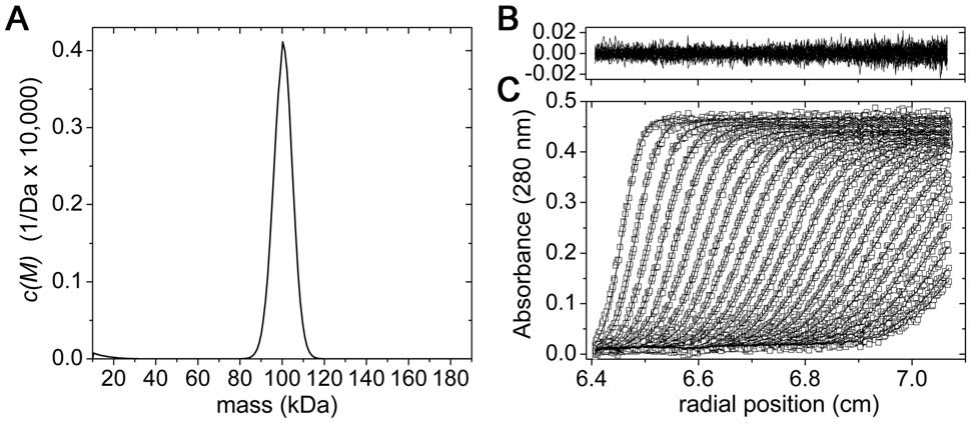
Sedimentation velocity analysis of *Cr*-DapL at 9.2 µM. A) Continuous mass, *c(M)*, distribution is plotted for *Cr*-DapL (solid line), suggesting a mass of ∼100 kDa. The predicted mass of the dimer is 97.66 kDa. Analysis was performed using the program SEDFIT [26], [27] at a resolution of 200, with mass_min_ = 10 kDa, mass_max_ = 180 kDa and at a confidence level (F-ratio) = 0.95. Statistics for the nonlinear least squares best fits were r.m.s.d. = 0.005, runs test-Z = 3. Residuals (B) for the *c(M)* distribution best fits (C) plotted as a function of radial position (cm) from the axis of rotation for *Cr*-DapL at 9.2 µM.

The continuous mass [*c(M)*]-distribution indicates that the recombinant *Cr*-DapL enzyme is dimeric in aqueous solution, with an apparent molecular mass of 100.2 kDa (Figure 5). The [*c(M)*]-distribution analysis also yielded an excellent fit, as indicated by the random distribution of residuals (Figure 5) and statistical parameters for the best-fit (r.m.s.d. = 0.005 and Runs test-Z score = 3.0). The frictional ratio (*f/f_0_*), which gives an indication of average shape in solution, was 1.51, suggesting that the hydrodynamic shape of *Cr*-DapL is asymmetric. These are the first data demonstrating that the enzyme DapL is dimeric in solution.

To examine the enzyme in atomic detail, we solved the crystal structure of *Cr*-DapL to 1.55 Å resolution. The enzyme crystallized in the space group *P*2_1_2_1_2_1_ and the structure was solved by molecular replacement using the *Arabidopsis thaliana* structure (*Ar*-DapL, PDB id: 2Z20 [12]), with two monomers in the asymmetric unit. The crystallization conditions and data collection details have been previously published [28], but are briefly described in the Materials and Methods section.

**Figure 6:**
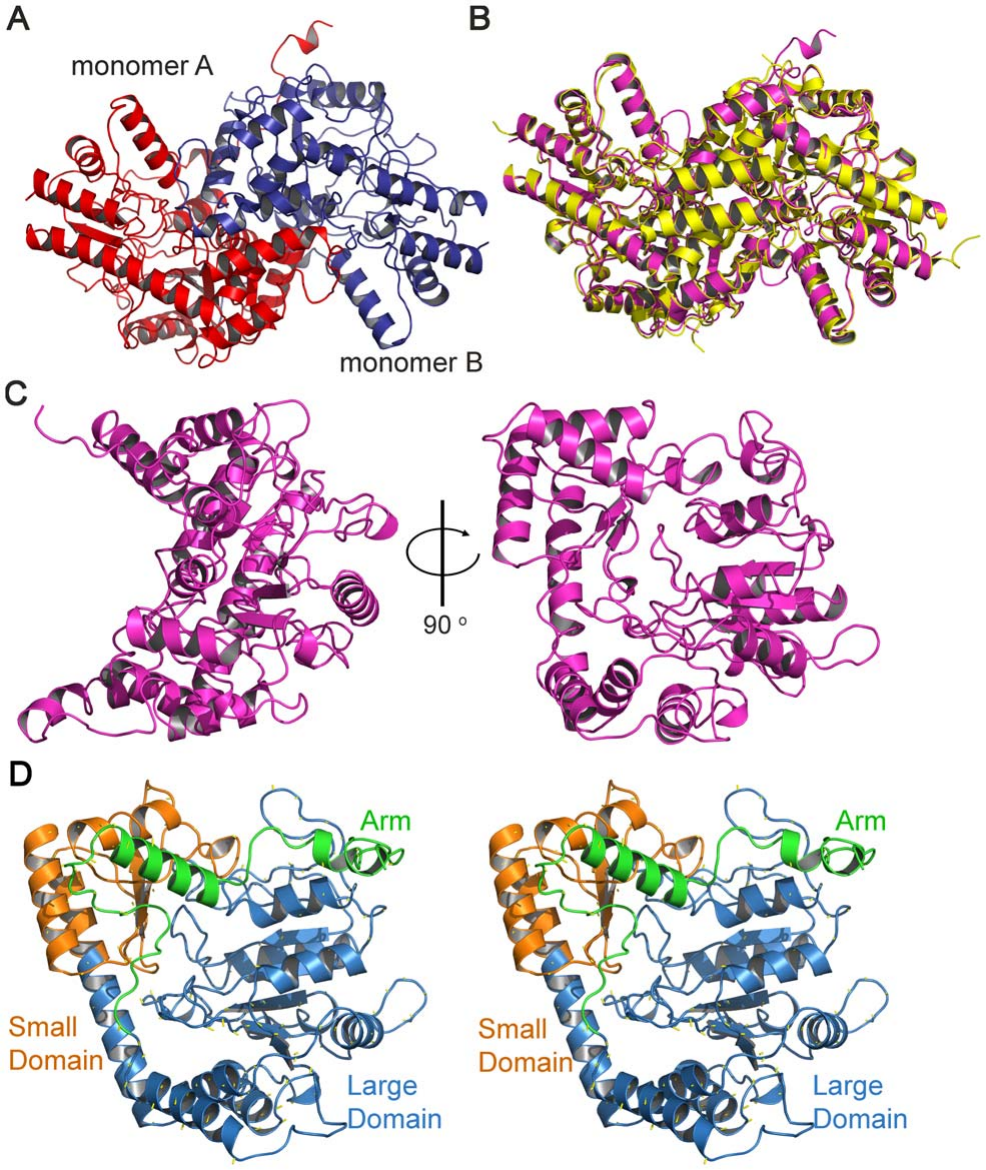
The crystal structure of *Cr*-DapL. A) The dimer in the asymmetric unit. This view looks down the non-crystallographic two-fold axis. B) An overlay of the *Cr-*DapL dimer (magenta) with that of the apo-*Ar-*DapL (3EI7, yellow). The r.m.s.d. for the overlay was 0.67 Å for the α-carbon atoms. C) Monomer structure with the domains highlighted in the stereo image (D).

Consistent with our sedimentation velocity experiment, the two monomers in the asymmetric unit interact closely to form a dimeric species (Figure 6A) and are related by a non-crystallographic two-fold symmetry axis. The interface between the monomers in the dimer buries ∼21% of the surface accessible area of each monomer and is composed primarily of hydrogen bonds, but also includes four salt bridges between residues R314 and D170, and residues D311 and R39 of each monomer. An overlay with the apo-Arabidopsis DapL dimer (PDB id: 3EI7 [13]) shows close agreement with an r.m.s.d. of 0.67 Å for 688 α-carbon atoms (Figure 6B).

A search for similar structural folds in the Protein Data Bank using the DALI server [29] revealed that, apart from the *Ar*-DapL, aspartate aminotransferases were the most closely related in structure to *Cr*-DapL. The most closely related structure was aspartate aminotransferase from *Pyrococcus horikoshii* (PDB id: 1GDE) with a r.m.s.d. of 2.4 Å for 365 α-carbon atom pairs. Another notable structure with significant similarity is *M. tuberculosis* enzyme *N*-succinyl diaminopimelate aminotransferase (PDB id. 2O0R [30], r.m.s.d. of 2.4 Å for 367 α-carbon atom pairs), which, interestingly, is also involved in lysine biosynthesis (*dapE* gene). *N*-succinyl diaminopimelate aminotransferase (DapC, Figure 1) mediates one of the three steps that bypassed by the reaction catalyzed by DapL. A previous phylogenetic analysis suggested that DapL was only distantly related to DapC enzymes, and indeed they share <20% sequence identity [4]. The finding that DapL and DapC show strong structural conservation may suggest a closer evolutionary link than first thought.

Each monomer is an α/β protein in a V-shaped conformation (Figure 6C) and is classified as a pyridoxal phosphate (PLP)-dependent transferase-like fold by SCOP. The monomers are largely α-helical in content, consistent with our CD data presented above. The electron density for the N-terminal residues was very poor and this likely contributes to the unordered structure (∼20%) predicted by the CD analysis. Given the high resolution, (1.55 Å), the electron density for the structure was clearly defined for most of the structure (see Supplementary Figure S2). The final model includes residues 33–439 in chain A and 26–438 in chain B. When the two monomers in the asymmetric unit were superimposed there was a very close agreement, with an r.m.s.d. of 0.15 Å for 339 α-carbon atoms. Based on the annotated domain structure of the Arabidopsis DapL model [12], the overall fold of each monomer of *Cr*-DapL consists of two domains, a large domain and a small domain (Figure 6D). The large domain (L83–E352) belongs to an αβ class, which folds into an α-β-α sandwich. The small domain (N26–P82 plus N353–G438) also belongs to the α-β class of protein fold and forms an α-β complex. In addition, the small domain also contains an “arm” region at the N-terminus.

**Figure 7:**
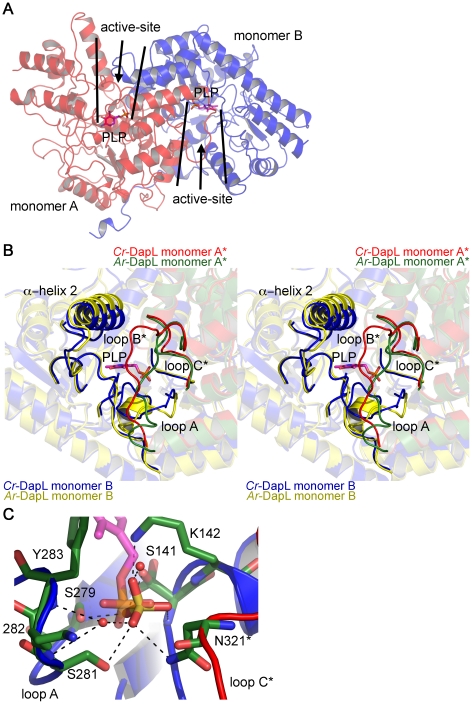
Location and orientation of the active-site of *Cr-*DapL. A) Location of the two active-sites in the dimer, as highlighted by the position of a PLP molecule, taken from an overlay of *Cr*-DapL with *Ar*-DapL+PLP structure (2Z20),shown in stick form (magenta). PLP was not found in the active site of *Cr-*DapL. B) Stereoview of the active-site showing the loops that contribute residues to the active-site. Again PLP is added to the structure from an overlay with *Ar*-DapL+PLP structure (2Z20). The image overlays the monomers of *Cr-*DapL (blue and red) with that of the apo-*Ar-*DapL (yellow and green). In B) and C), the asterisk emphasizes loops that are contributed from the opposing monomer in the dimer. C) Bonding of residues in loops A and C* with the sulfate, which sits in the same position as the phosphate of PLP.

Based on an overlay of the *Cr*-DapL structure with that of the Arabidopsis structure bound to PLP (PDB id. 2Z20), the active site sits in a crevice between the two lobes of the V-shaped monomer and is lined with residues from both monomers in the dimer (Figure 7A). This suggests a functional reason for the observed dimeric structure. It has previously been noted that this is a common quaternary structure for aminotransferases [12], [31].

The geometry of the active site is quite different when compared to apo-*Ar*-DapL, despite conservation of many of the residues responsible for substrate and cofactor binding (Supplementary Figure 3A and B). An overlay of the active site with that of the apo-*Ar*-DapL structure (PDB id. 3EI7 [13]) shows key differences in the orientation of loops A and B within the active site and a displacement of α-helix 2 (Figure 7B), and is evident in the structural alignment presented in Supplementary Figure S3B.

In the *Ar*-DapL structure, loop A contains a short α-helix, which is involved in co-factor binding, including a key *l*-lysine residue (K270, *Ar*-DapL numbering) that covalently binds PLP to form the reactive aldimine cofactor. In the *Cr-*DapL structure, this loop, which comprises residues F280–G292 and includes the equivalent key *l*-lysine residue (K282, *Cr*-DapL numbering), adopts a random configuration (Figure 7B). We note, however, that our crystals grew in the presence of LiSO_4_ (200 mM) and the structure contains a sulfate ion very close to where the phosphate of PLP might sit in the active-site (Figure 7C). The sulfate makes direct hydrogen bonds to the side-chain and main-chain atoms of residues in loop A, as well as two water bridging interactions, including K282. This perhaps explains the altered conformation of the loop and, given the sulfate sits in nearly the identical place as the phosphate of PLP, suggests the PLP binding conformation may be different when compared to *Ar*-DapL.

Loop B, which comprises residues A99–G114, also adopts a different configuration to the equivalent loop in *Ar*-DapL and this may in part be responsible for the displacement of α-helix 2. Loop B, which sits at the top of the active-site of the opposing monomer, is thought to act as a gate to the active-site for substrates [13]. The high temperature factors (B-factors) for this loop (Supplementary Figure S4C and D) suggest that it is flexible, presumably allowing substrates access to the active-site even though it occludes the entrance. Increased flexibility in this loop was also observed for the apo-*Ar-*DapL structure [13]. In a series of ligand bound *Ar-*DapL models, Watanabe *et al.* have found that this loop becomes ordered when substrates are bound, preventing access to the active site [13]. In our *Cr-*DapL model, loop B, although flexible, also interacts with the displaced α-helix 2 *via* a water-bridging hydrogen bond between the main-chain atoms of Y107 and A56, and a hydrogen bond between R59 and S105. In addition, the N-terminal end of loop B binds to a second sulfate situated at the entrance of the active-site in each monomer, with hydrogen bonds to residues R101 and Y104. We also note that loop B is considerably shorter in the four aspartate aminotransferases and *Mtb*-succinylDAP aminotransferase most closely related to *Cr-*DapL (∼10 residues long compared to 15 residues in *Cr-*DapL), allowing unobstructed access to the active-site cleft (see Supplementary Figure S5). In addition, the α-helix preceding loop B is also longer by ∼1 full turn in the DapL enzymes compared to the five closely related aspartate aminotransferases (Supplementary Figure S5).

Another difference is that loop C, which comprises residues T318–N325, is significantly disordered in the apo-*Ar-*DapL structure, but well-ordered in our structure. This may again be due to a hydrogen bond (2.9 Å) between the side-chain of N321 within loop C with the ordered sulfate in the active-site (Figure 7C).

**Figure 8:**
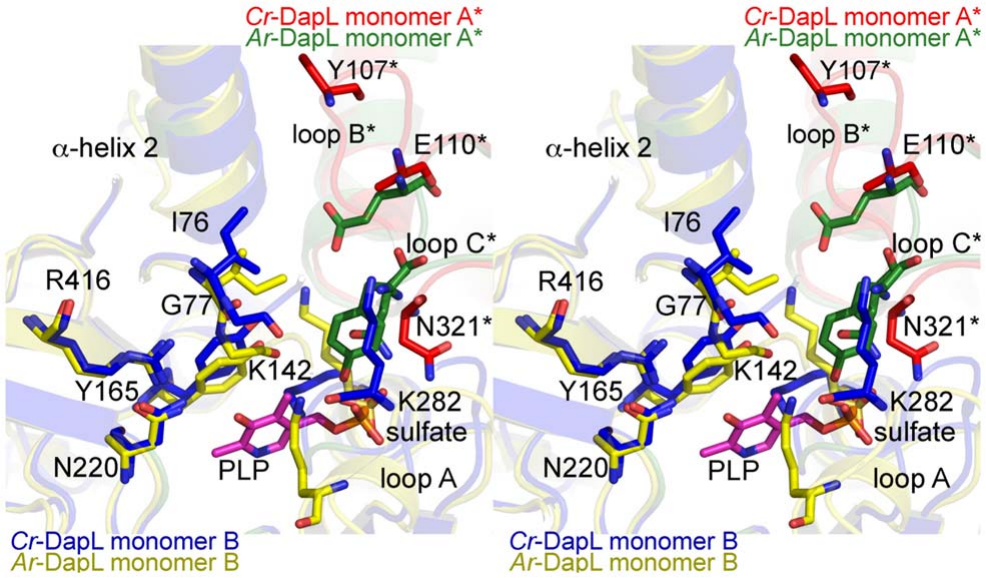
Overlay active site residues of *Cr-*DapL with apo-*Ar*-DapL. Stereoview of the putative active-site residues conserved between *Cr*-DapL and *Ar-*DapL (see sequence and structural alignments in Supplementary Figure 3). As in Figure 7, the asterisk emphasizes residues that are contributed from the opposing monomer in the dimer. Numbering is based on the *Cr*-DapL structure. The sulfate and PLP molecules are also shown.

The altered loop structures in the *Cr*-DapL active-site, compared to the apo-*Ar*-DapL model (Figure 7B), led to a number of putative catalytic side-chains adopting alternate conformations (Figure 8) and suggests that a major reorientation of the active site is necessary upon cofactor and substrate binding. Figure 8 shows the active site residues putatively responsible for substrate binding and catalysis. The major differences surround the loop A, where K282 is reoriented relative to the apo-*Ar*-DapL. In loop B, Y107 is facing out of the active site compared to the equivalent residue in apo-*Ar*-DapL, which points into the active active-site. K142, which is thought to be necessary for substrate recognition, fills the space that would be taken by the PLP cofactor. E110 from the other monomer in the dimer and N321, which are both involved in substrate recognition, are roughly in the same position.

To summarize our structural studies, we have shown by AUC that *Cr-*DapL is a dimer in solution. The enzyme is also dimeric in the crystalline form. *Cr*-DapL is an α/β protein with each monomer of the dimer adopting a PLP-dependent transferase-like fold in a V-shaped conformation. CD data is consistent with proportions of secondary structure found in the crystal structure, suggesting it is similarly folded in solution. The active site is situated in a crevice between the two lobes of the V-shaped monomer and comprises residues from both monomers in the dimer. There is some rearrangement of the active site residues when compared to the apo-*Ar*-DapL structure, although the putative catalytic residues are conserved, suggesting that cofactor and substrate binding requires reorientation of these residues.

### The essentiality of DapL in Arabidopsis

Since it is difficult to show gene essentiality in the alga *C. reinhardtii*, we chose to investigate whether *dapL* was an essential gene in the plant model organism *Arabidopsis thaliana*. Embryo lethality screening can be used to assess the essentiality of a particular gene and has identified genes that are essential in other amino acid biosynthetic pathways, including histidine [32]. One of the characteristics of this technique is that aborted seeds can be observed in the fruit of mutant plants. DapL was previously annotated as an aminotransferase-like enzyme designated Aberrant Growth and Death 2 protein and was shown to be essential for plant development *via* a T-DNA insertion mutant in the first exon of the gene [33]. However, it is plausible that the phenotype observed by Song *et al.* is a direct result of having multiple T-DNA insertions, which occur at a significant rate in Arabidopsis [34]. Thus, we used embryo lethality screening to carefully test the hypotheses that DapL was essential in Arabidopsis.

**Figure 9:**
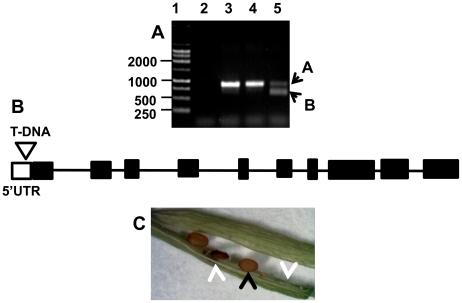
Analysis of T-DNA mutant line SAIL_208_H11. A) PCR analysis of the SAIL_208_H11 Arabidopsis T-DNA mutant line: Lane (1)–DNA ladder (base pairs), Lane (2)-negative control, Lane (3)-WT-non transgenic plant, Lane (4)-WT-segregant, Lane (5)-heterozygous plant. B) Schematic localization of the T-DNA insertion site, which is located in the 5′ UTR of the gene. C) Phenotype analysis of a heterozygous silique showing the WT seed (black arrow) and mutant or aborted seeds (white arrows).

Analysis of a different T-DNA insertion in the *dapL* gene from Arabidopsis (SAIL_208_H11) from our studies show for the first time that *dapL*, which is now known to be involved in *l*-lysine biosynthesis, is an essential gene in plants and possibly in other photosynthetic cohorts. This assay was carried out using a PCR strategy to identify a heterozyogous plant along with a wild type segregant from the mutant line (Figure 9A). The amplicon corresponding to the T-DNA insertion site; the lower band in lane 5 denoted as (B) was excised from the gel and subjected to nucleotide sequencing. Nucleotide sequencing confirmed that the T-DNA is located in the promoter region of the gene, 300 base pairs upstream of the initiation start codon (Figure 9B). The heterozygous plant that was identified in the PCR analysis was further grown to maturity and the siligues were observed for the mutant phenotype. The black arrow shows the phenotype of a wild type seed while the white arrows show the phenotypes of mutant embryos (Figure 9C). Due to the essentiality of the gene, homozygous plants were not observed using this strategy.

The phenotype analysis confirms that *dapL* is an essential gene in Arabidopsis by the observation of aborted embryos and undeveloped embryos in the fruit of the plant (Figure 9C). Given that the *dapD*, *dapC* and *dapE* genes are absent from the Arabidopsis and algae genome, and that corn, tobacco, *Chlamydomonas* and soybean do not show DapC or DapE activity in lysates [35], our results strongly suggest that the DapL pathway is the only route to *l*-lysine in these photosynthetic systems. Given that we now have shown that *dapL* is an essential gene in Arabidopsis, it is plausible that this is a general feature in photosynthetic cohorts, including algae. If this is the case, the identification and characterization of the DapL ortholog from the algae *C. reinhardtii*, including our kinetic and structural studies, provides useful information with respect to algaecide development.

From an evolutionary perspective, we think that the acyl and Ddh pathways evolved to allow for faster growth rates. This assertion is supported by the observation that organisms that use the DapL pathway for DAP/lys synthesis are known to grow significantly slower than organisms that contained either the acyl pathways or a combination of the acyl and Ddh pathways. Structural data supports the idea that the substrate for the DapL enzyme is the acyclic keto form of THDPA, since the active site does not easily fit the cyclic THDPA species [12], [13]. For a transamination reaction to be catalyzed by DapL, the system relies on the spontaneous opening of THDPA that would expose the keto group. We note that theequilibrium between the cyclic and acyclic form of the species has not been elucidated [36]. The function of DapD is to add an acyl protecting group to THDPA, which results in the transition of THDPA from the cyclic form to the acyclic form. This transition exposes the keto group for a transamination reaction catalyzed by DapC. Since lysine and DAP are components of cell wall, one would expect that DAP/lys synthesis is coordinated to the growth of the organism.

In conclusion, an *in vivo* and *in vitro* characterization of the DapL ortholog from the alga *C. reinhardtii* reveals that the enzyme could functionally complement the *E. coli dap* auxotrophs and was essential for plant development in Arabidopsis. The recombinant enzyme was able to inter-convert THDPA and *l,l-*DAP, showing tight substrate specificity. The structure of *Cr*-DapL was solved in its *apo* form, showing an overall architecture of a α/β protein with each monomer in the dimer adopting a PLP-dependent transferase-like fold in a V-shaped conformation. The active site comprises residues from both monomers in the dimer and show some rearrangement when compared to the DapL structure from Arabidopsis. Finally, the quaternary structure was shown to be dimeric at the concentrations tested. Since animals do not possess the enzymatic machinery necessary for the *de novo* synthesis of *l*-lysine, enzymes involved in this pathway are attractive targets for the development of antibiotics, herbicides and algaecides.

## Materials and Methods

### *C. reinhardtii* growth conditions

*C. reinhardtii* strain CC-1690 was obtained from *Chlamydomonas* Genetics Center (Duke University, Durham, NC) and was grown in Tris-Acetate-Phosphate (TAP) medium. The strain was grown in a growth chamber with a 16 hour light and 8 hour dark period for 7 days. The temperature was 24°C during the light period and 20°C during the dark. The light intensity was approximately 120 µE M^−2^ sec^−1^.

### Functional complementation plasmid construct

The cloning of the full length dapL cDNA from *C. reinhardtii* was previously reported by us [28]. Briefly, the cDNA was cloned into a pET30a vector to give the pET30a+*Cr*-DapL plasmid, which gave a hexa-histidine and S-TAG epitope derived from pET30a plasmid at the amino terminus. The plasmid used for functional complementation of the *E. coli dapD/E* double mutant was produced by sub-cloning the *XbaI* and *HindIII* fragment from pET30a+*Cr*-DapL into pBAD33, to give pBAD33+*Cr*-DapL. The fusion protein produced from the pBAD33 construct is identical to the protein produced from the pET30a construct.

### Functional complementation of *dapD/dapE E. coli* mutant

The *E. coli* mutant AOH1 (*ΔdapD::Kan2,dapE6*) [3] was transformed with pBAD33 or pBAD33-*Cr*-DapL and grown on LB agar medium supplemented with 50 µg mL^-1^ DAP and 34 µg mL^−1^ chloramphenicol and 50 µg mL^−1^ kanamycin . Individual colonies were then replica plated onto LB medium plus 0.2% (^w^/_v_) arabinose with or without 50 µg mL^−1^ DAP. The cultures were grown at 30 °C for 24 hours.

### T-DNA mutant analysis

The *Arabidopsis thaliana* T-DNA mutant line SAIL_208_H11 was obtained from the Arabidopsis Biological Resource Center (ABRC) (http://abrc.osu.edu/). For T-DNA insertion analysis, the zygosity was assessed by PCR amplification using the RED Extract-N-Amp™ Plant PCR kit following the manufacturer's protocol (Sigma Inc., St. Louis, MO, USA). A PCR strategy using three primers was employed using the 12 picomoles of each primer. The gene specific primers were 5′-AAGAAAACAAAACGACGCACC-3′ and 5′-TTGGATGAAGCAAAGTCTGTCAAC-3′ and the T-DNA specific primer was 5′-GCCTTTTCAGAAATGGATAAATAGCCTTGCTTCC-3′. The following PCR conditions was used in the PCR assay: 1 cycle at 94°C for 3minutes followed by 30 cycles at 94°C for 30 seconds, 60°C for 30 seconds, 72°C for 2 minutes. The PCR amplicons were resolved on 0.8% (^w^/_v_) agarose gel.

### Protein expression and purification

Full details for the expression and purification of *Cr*-DapL are described elsewhere [28]. In summary, the plasmid pET30a+*Cr*-DapL was transformed into *E. coli* BL21-CodonPlus-RIPL strain and was grown in LB broth. Protein expression was induced with IPTG for 4 hours at 25°C, followed by sonication in a solution of 50 mM sodium phosphate (pH 8.0) and 300 mM NaCl. The extract was incubated with Talon Metal Affinity Resin for 30 minutes at 4°C and extensively washed with sonication buffer containing 10 mM imidazole pH 8.0, followed by elution with sonication buffer containing 250 mM imidazole. The pure protein was concentrated in an Amicon Ultra 10 kDa Mw cutoff filter unit, exchanging the buffer with 100 mM HEPES-KOH containing 1 mM DTT and 2 mM EDTA (pH 7.6). To remove any precipitated protein prior to crystallization, the purified protein was passed through a S200 size exclusion column pre-equilibrated with buffer (20 mM Tris.HCl, 5 mM DTT, 2 mM EDTA, pH 7.8), followed by concentration with an Amicon Ultra 10 kDa Mw cutoff spin filter unit.

For expression of *Corynebacterium glutamicum meso-*DAP dehydrogenase (Ddh), *E. coli* BL21 (DE3) harboring the plasmid pET28+CgDdh was grown in LB broth containing 50 µg mL^−1^ kanamycin at 37°C to an OD_600_ of 0.5. Ddh expression was induced with 0.5 mM IPTG for 4 hours at 25°C. The cells were lysed by sonication in 100 mM HEPES-KOH (pH 7.6). The protein was concentrated using an Amicon Ultra 10 kDa Mw cutoff device. The Ddh enzyme comprised approximately 90% of the soluble fraction and was not further purified. For long-term storage, the enzyme was stored in 50% glycerol.

### Enzyme assays

Three different assays were used to measured *L,L*-DAP aminotransferase activity; two measured the synthesis of THDPA and another measured the production of *L,L*-DAP synthesis. The first assay measured the formation of THDPA using *ortho*-aminobenzaldehyde, which forms dihydroquinazolium and absorbs light at 440 nm. A second assay used *meso*-DAP dehydrogenase coupled to THDPA synthesis by measuring the oxidation of NADPH. A third assay measured the physiologically significant forward reaction using 2-oxoglutarate dehydrogenase coupled to 2-oxoglutarate synthesis from THDPA by measuring the oxidation of thio-NAD**^+^**.

#### Measurement of l,l-diaminopimelate aminotransferase: the 2-aminobenzaldehyde (OAB) assay

The 2-aminobenzaldehyde (OAB) assay contained in 0.5 mL 100 mM HEPES-KOH (pH 7.6), 0.5 mM amino donor, 2 mM 2-oxoglutarate, and 1.25 mM OAB and 10.0 µg of pure recombinant *Cr*-DapL protein. Reactions were incubated at 30°C and the change in absorbance was measured continuously at 440 nm with a DU 640 spectrophotometer (Beckman Coulter, Brea, CA, USA).
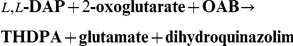



#### Measurement of L,L-diaminopimelate aminotransferase: the two enzyme system

Quantitative assays of the physiological reverse activity was measured in 0.5 mL 100 mM HEPES-KOH (pH 7.6), 0.3 mM NADPH, 50 mM NH_4_Cl, 0.5 mM *L,L*-DAP, 5 mM 2-oxoglutarate, 4.0 µg *Cg*-Ddh, and 4.0 µg of pure recombinant *Cr*-DapL produced from the pET30a-*Cr*-DapL construct. The reactions were incubated at 30°C and the decrease in absorbance of 340 nm was monitored.
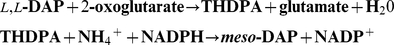



#### Measurement of l,l-diaminopimelate aminotransferase: the three enzyme system

Quantitative assay for the physiologically relevant forward direction was measured in 0.5 mL containing 100 mM HEPES-KOH (pH 7.6), 0.5 mM NADP, varying amount of *meso-*DAP, 0.3 mM thio-NAD, 0.3 mM CoA, 5.0 mM glutamate and 8.0 µg *Cg*-Ddh. The reactions were run to completion (30 minutes), determined by measuring the absorbance at 340 nm. The wavelength of spectrophotometer was changed to 398 nm followed by the addition of 200 µg of 2-oxoglutarate dehydrogenase (Sigma Inc., St. Louis, MO, USA) and 8.0 µg of pure recombinant *Cr*-DapL. Thio-NADH production was measured by the increase in absorbance at 398 nm over a 30 minute time span.
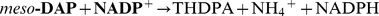


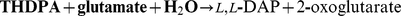


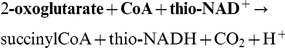



### Circular Dichroism (CD)

Spectra were collected between wavelengths of 190 and 240 nm in a Jasco J-815 CD spectrometer at 20°C using a 1 mm path length quartz curvette, 1 nm step size, 1 nm bandwidth, and 2 s averaging time. Spectra of *Cr*-DapL in 10 mM Tris-HCl, 100 mM KCl pH 8.0 were recorded at a protein concentration of 1 µM. CD spectra were analyzed by non-linear least-squares regression using the CONTIN algorithm and various reference databases available with the CDPro software package (available from http://lamar.colostate.edu/~sreeram/CDPro/main.html) [37].

### Analytical ultracentrifugation (AUC)

AUC experiments were conducted in a Beckman model XL-I instrument at 20 °C. The protein sample (*Cr*-DapL, 0.45 mg mL^−1^, 9.2 µM, monomeric mass = 48,830 kDa, v-bar = 0.721 mL g^−1^) was buffer exchanged with 50 mM HEPES, 0.5 mM DTT, 1 mM EDTA, 50 mM NaCl pH 8.0) and loaded into double sector quartz cells and mounted in a Beckman 4-hole An-60 Ti rotor. Solvent density (1.00435 g ml^−1^ at 20°C), viscosity (1.0341 cp) and an estimate of the partial specific volumes were computed using the amino acid composition and the program SEDNTERP [38].

For the sedimentation velocity experiments, 300 µl of sample and 320 µl of reference solution were centrifuged at a rotor speed of 45,000 rpm, and the data was collected at a single wavelength (280 nm) in continuous mode, using a time interval of 0 s and a step-size of 0.003 cm without averaging. The absorbance versus radial position profiles were used in the nonlinear least squares analysis. Initial scans were eliminated from the nonlinear regression analyses due to temperature fluctuations at the beginning of the experiment. Sedimentation velocity data at multiple time points were fitted to a continuous sedimentation-coefficient model using the program SEDFIT [26], [27], available from http://www.analyticalultracentrifugation.com).

### Macromolecular crystallography

X-ray diffraction data from crystals of *Cr-*DapL were collected on the MX2 beam-line at the Australian Synchrotron (Clayton, Australia). Details for the protein crystallization and data collection have been published elsewhere [28], but briefly, crystals were obtained at 293 K by mixing 150 nL of protein solution (8.6 mg mL^−1^, in 20 mM Tris.HCl, 5 mM DTT, 2 mM EDTA, pH 7.8) and 150 nL of reservoir solution (200 mM lithium sulfate, 25% *w*/*v* polyethylene glycol 3350, 100 mM Bis-Tris propane, pH 5.5, including 0.02% *w/v* sodium azide) using the sitting drop method. Diffraction data sets were processed, scaled, and merged using the package MOSFLM [39] and SCALA [40]. Molecular replacement (PHASER [41]) was used to solve the initial phases with the Arabidopsis DapL structure (PDB id 2Z20) as a search model. Restrained refinement was performed using REFMAC5 [40] or PHENIX.REFINE [42]with iterative model building using COOT [43]. A round of simulated annealing, using PHENIX.REFINE [42], was included early in the refinement scheme.

We chose to refine the final model anisotropically, even though a resolution of 1.55 Å is in the grey zone for refining protein structures in this way, given the low ratio of “observations” to parameters that are refined [44]. We justify this strategy thus. 1) The ratio of “observations” to refined parameters (*N*/p) is sufficient (*N*/p = ∼2.3 = (122,383 reflections+∼27,290 restraints) / 65,070 parameters (see [45] and references therein)) and is roughly comparable to the same structure refined isotropically at 2.19 Å resolution (*N*/p = ∼2.5 = (43,942 reflections+∼27,290 restraints) / 28,920 parameters). 2) We note that other structures close to or at lower resolution have also been refined anisotropically (see Table 1 in [44]). 3) The restrained refinement, employing the default restraints in REFMAC5.5_0.102, was stable. 4) An analysis by the online Protein Anisotropic Refinement Validation and Analysis Tool (PARVATI, [44]) showed a reasonable distribution in anisotropic protein atoms, with a mean of 51 and a σ of 13 (Supplementary Figure S1A). 5) There were no non-positive definite atomic displacement parameters in the model. And 6), there was a 2.1% drop in the *R*_free_ statistic, from 19.1% to 17.1%, and a similar drop in *R*_fact_, from 15.7% to 14.2%, suggesting that expanding the model to include anisotropy represents a better fit to the data.

The electron density for the N- and C-terminal residues was very poor; thus, the final model includes residues 33–438 in chain A and 26–438 in chain B. Residues 105–110 of both monomers, which comprise loop B at the entrance of the active site of the opposing monomer, were also poorly defined and therefore tight NCS restraints for this region were included throughout the refinement to stabilize the geometry of the loop. Side-chain atoms without electron density to guide model building were deleted from the final model. The model also included four sulfate ions, two glycerol molecules, and three azide molecules. The structure was validated using the MolProbity server (http://molprobity.biochem.duke.edu/) [46]. The Ramachandran plots (Supplementary Figure S6) showed that 99.9% of the residues in the model were in the most favoured or additionally allowed regions. Refinement statistics are shown in Table 4. The final model has been deposited in the Protein Data Bank (PDB id 3QGU).

## Supporting Information

Figure S1 Michaelis-Menten plots of the four substrates that were used in the kinetic assays. The plots were drawn using GraphPad Prism v 3.03.
(TIF)

Figure S2 A) Electron density surrounding the sulfate in the active site of *Cr*-DapL. B) Electron density about the α-helix 2, close to the active site. In both A) and B), the 2F_o_-F_c_ electron density is displayed in blue and contoured to 1 σ. The F_o_-F_c_ density is also displayed in red (contoured to -3 σ) and in green (contoured to 3 σ).
(TIF)

Figure S3 A) Sequence alignment of *Cr*-DapL with Arabidopsis DapL generated using the ClustalW server (http://www.ebi.ac.uk/Tools/msa/clustalw2/). Putative active-site residues, based on the structural studies of the ligand bound *Ar*-DapL enzyme [12], [13], are shown in red. The loop regions correspond to the active site-loop regions shown in Figure 8B, where the * refers to the loop contributing to the active site of the opposing monomer. B) Structural alignment of the apo-*Cr*-DapL structure reported here with the apo-*Ar*-DapL structure (PDB id 3EI7). The alignment was conducted using the program STRAP (http://3d-alignment.eu/) [47].
(TIF)

Figure S4 Anisotropic model of *Cr*-DapL. A) A plot of the distribution of anisotropy for the protein, ligand and water atoms. B) Thermal ellipsoids of *Cr-*DapL structure, colors show atoms with high B-factors (red) and low B-factors (blue). C) Cartoon representation of dimer again showing regions with relatively high B-factors. D) Plot of B-factors per residue for chain A (black) and chain B (red).
(TIF)

Figure S5 Comparing the conformation of Loop B in the DapL and closely related aspartate aminotransferases. The aspartate aminotransferases are shown in grey and the *Cr*-DapL is in red. PLP is shown to highlight to position of the active- site relative to Loop B and is taken from the structure of the Arabidopsis structure (teal, PDB id. 2Z20). The five closely related aminotransferase structures (as determined by the DALI server and shown in grey) are: *Thermus thermophiles* aspartate aminotransferase (PDB id. 1B5P); *M. tuberculosis N-*succinyl diaminopimelate aminotransferase (PDB id 2O0R); *Phormidium lapideum* aspartate aminotransferase (PDB id. 1J32); *Pyrococcus horikoshii* aspartate aminotransferase (PDB id. 1GDE); *Thermotoga maritima* aspartate aminotransferase (PDB id. 1O4S).
(TIF)

Figure S6 Ramachandran analysis of *Cr*-DapL model. As analyzed by the MolProbity server [46]. The single residue, Thr34, that lies outside the allowed regions of the Ramanchandran plot had rather weak density accounting for its unusual main-chain geometry.
(TIF)
