# Supplementary figures and images for: L,L-Diaminopimelate Aminotransferase from Chlamydomonas reinhardtii: A Target for Algaecide Development
Source: PLoS One. 2011 May 25;6(5):e20439. doi: 10.1371/journal.pone.0020439 (PMC3102117; doi:10.1371/journal.pone.0020439)

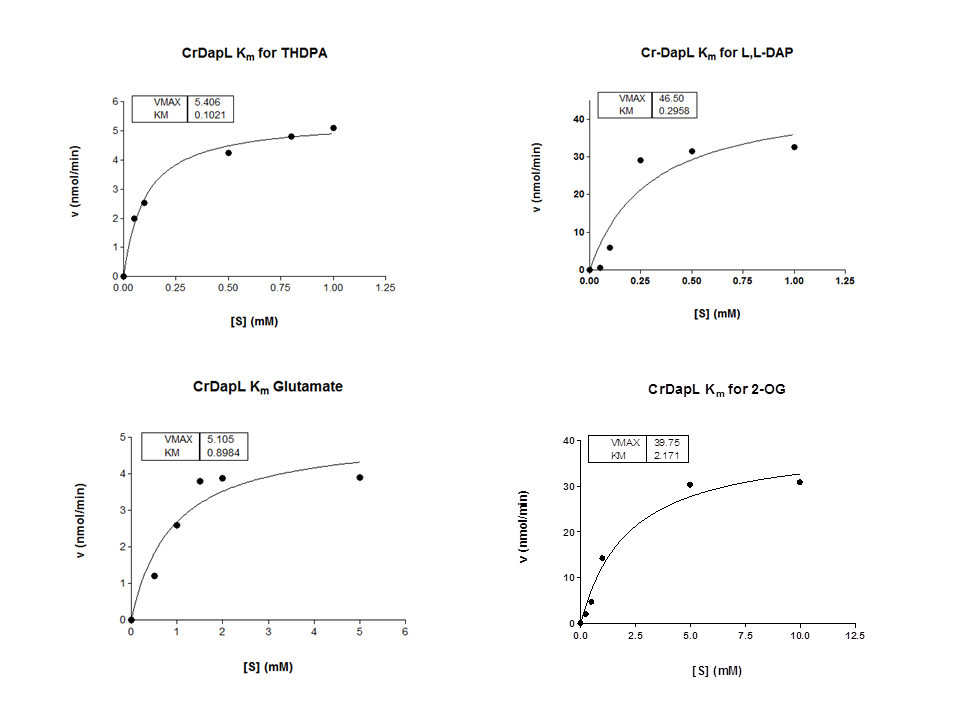

Supplement: Figure S1 — Michaelis-Menten plots of the four substrates that were used in the kinetic assays. The plots were drawn using GraphPad Prism v 3.03. (TIF) [file pone.0020439.s001.tif]

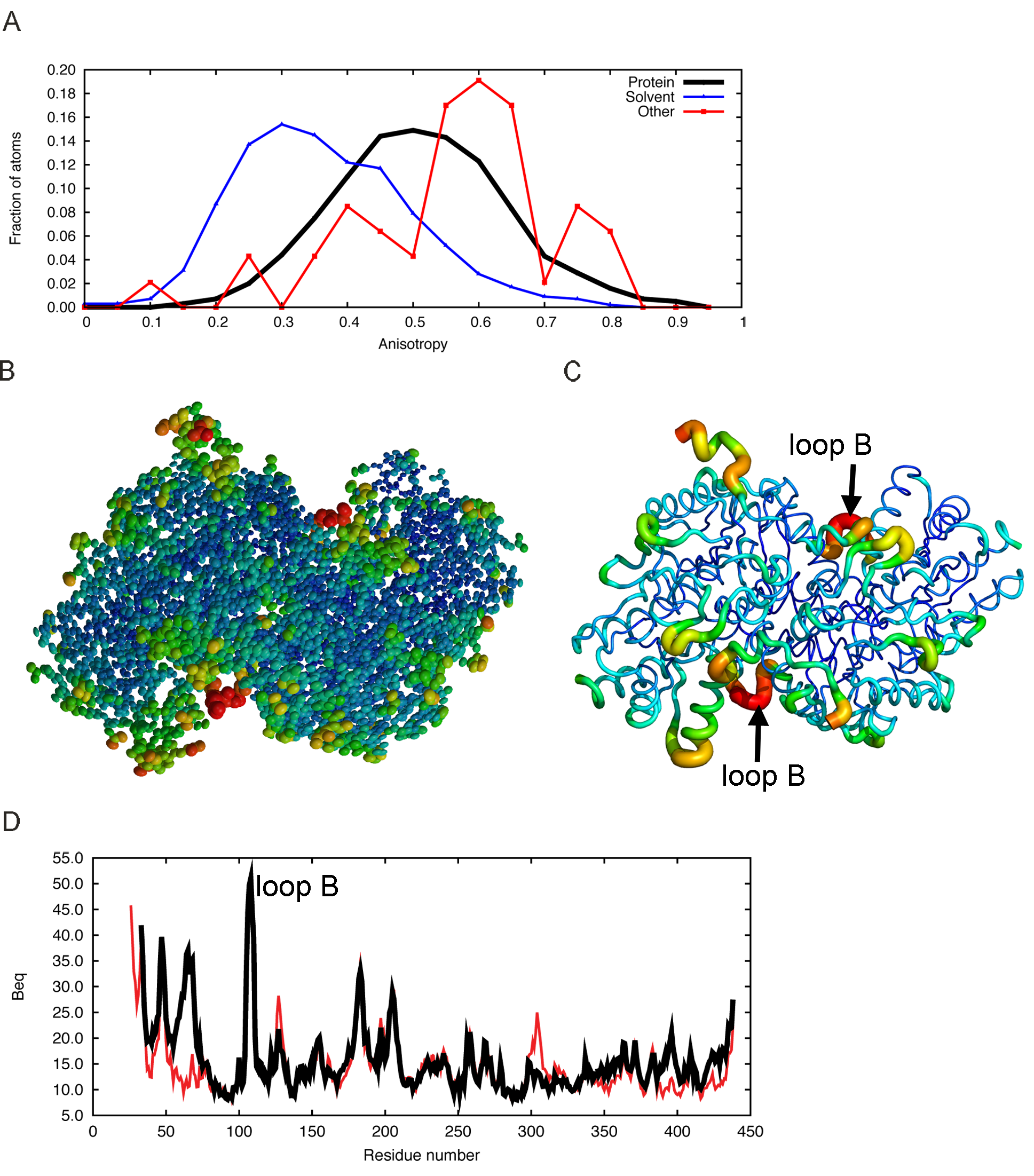

Supplement: Figure S4 — Anisotropic model of Cr-DapL. A) A plot of the distribution of anisotropy for the protein, ligand and water atoms. B) Thermal ellipsoids of Cr-DapL structure, colors show atoms with high B-factors (red) and low B-factors (blue). C) Cartoon representation of dimer again showing regions with relatively high B-factors. D) Plot of B-factors per residue for chain A (black) and chain B (red). (TIF) [file pone.0020439.s004.tif]

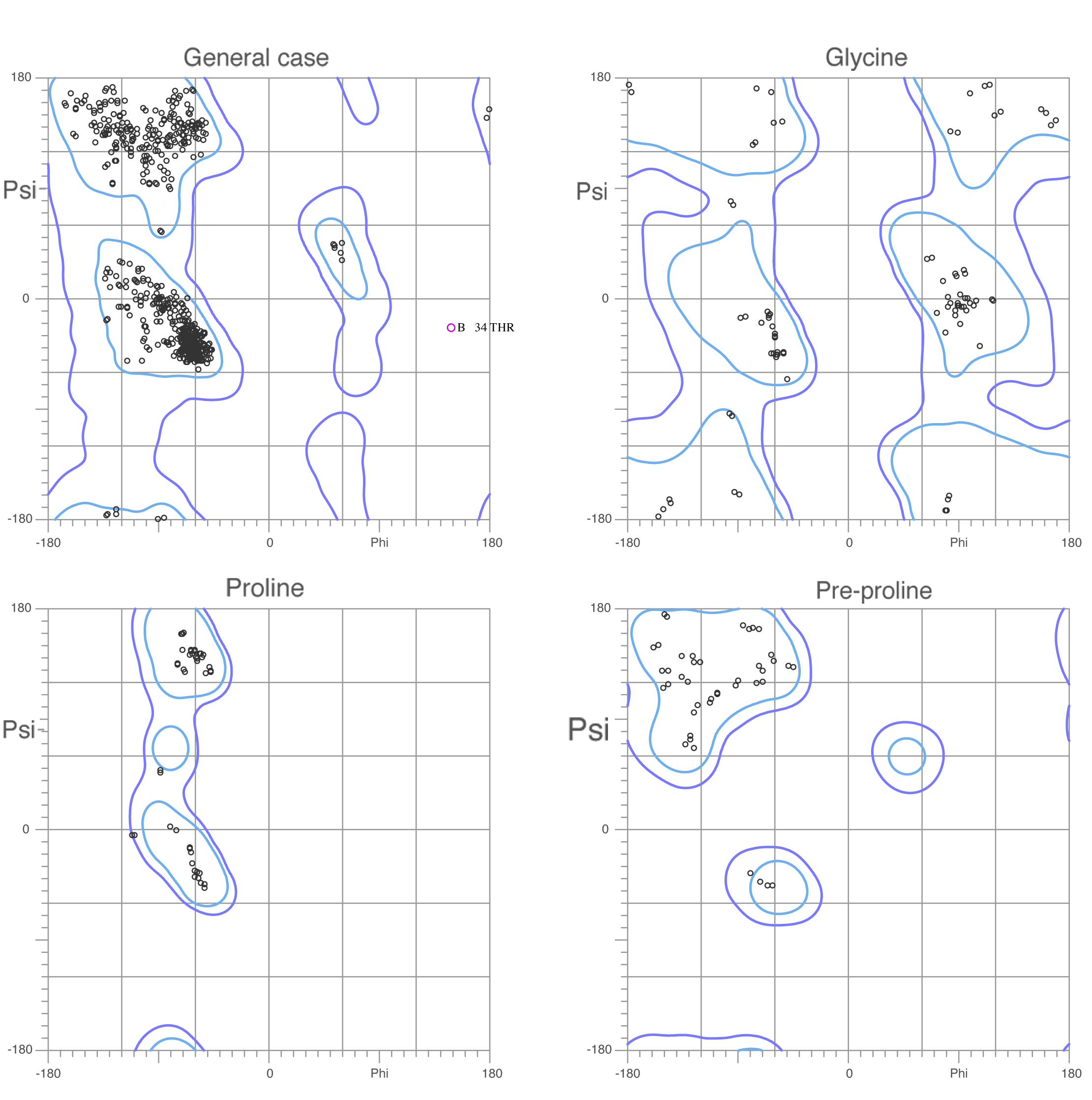

Supplement: Figure S6 — Ramachandran analysis of Cr-DapL model. As analyzed by the MolProbity server [46]. The single residue, Thr34, that lies outside the allowed regions of the Ramanchandran plot had rather weak density accounting for its unusual main-chain geometry. (TIF) [file pone.0020439.s006.tif]
